# Dampening Effects of Perceived Teacher Enthusiasm on Class-Related Boredom in College Students: Longitudinal Mediation Effects of Perceived Task Value

**DOI:** 10.3389/fpsyg.2021.712441

**Published:** 2021-09-16

**Authors:** Guan-yu Cui, Jing-yi Chen, Chen Wang, Chen Zhang, Xia Zhang, Yun-jun Hu

**Affiliations:** ^1^Department of Psychology, School of Education, Wenzhou University, Wenzhou, China; ^2^Research Center for Psychology and Behavior, Wenzhou University, Wenzhou, China; ^3^Center for Brain, Mind and Education, Shaoxing University, Shaoxing, China; ^4^School of Teacher Education, Shaoxing University, Shaoxing, China; ^5^Department of Nursing, Henan Medical College, Zhengzhou, China; ^6^Department of Students’ Affairs, Wenzhou University of Technology, Wenzhou, China

**Keywords:** teacher enthusiasm, task value, class-related boredom, longitudinal mediation effects, dampening effects

## Abstract

This study aims to explore the longitudinal mediation effects of college students’ perceived task value (PTV) between perceived teacher enthusiasm (PTE) and class-related boredom (CB). We conducted a longitudinal survey among college students from five colleges at the second (T1), sixth (T2), tenth (T3), and fourteenth week (T4) in a semester, and overall 1,371 students completed all the measurements. In the survey, a battery of questionnaires was used to measure the students’ PTE, perception of task difficulty, perception of task value, and CB. At T1, boredom proneness was measured as a control variable. Analysis of the longitudinal data showed that after controlling for the effects of boredom proneness and perceived task difficulty, students’ PTE was a significant predictor of CB, and students’ PTV played a significant mediating role in this causation relationship. The study supported the importance of the control-value theory in explaining the mitigating effect of students’ PTE on CB, especially highlighting the role of PTV.

## Introduction

Although teacher enthusiasm is recognized as the core index of affecting the quality of teaching, teacher enthusiasm may play a more important role than what we have known, especially on students’ academic emotions (Keller et al., 2014, 2016). Previous literature has shown that teacher enthusiasm is associated with low levels of students’ class-related boredom (CB; Cui et al., 2017a, 2020; Wang et al., 2020) and students’ perceived task value (PTV) may play an intermediary role between them (Cui et al., 2017b). To date, few longitudinal studies have examined the causal relationships among these variables. Compared with a cross-sectional design that cannot reveal causality, a longitudinal design is more rigorous in providing reliable evidence. This study intends to use a longitudinal research design to examine the predictive effect of students’ perceived teacher enthusiasm (PTE) on their classroom boredom and the mediating role of task value in this causality relationship.

## Literature Review

### Class-Related Boredom

Boredom, a negative and low-arousal emotion that is widely experienced by individuals in many situations (Chan et al., 2018; Martz et al., 2018; Sharp et al., 2019; Schwartze et al., 2020; Tam et al., 2021), was once thought to be a human “plague” (Pekrun et al., 2010), explained by the expressions “boredom like hell” (Maher et al., 2018) and “bored to death” (Britton and Shipley, 2010). In teaching and learning situations, boredom is typically experienced in class, and can be referred to as CB, which has a significant negative impact on students’ learning processes and learning outcomes (Harris, 2000; Pekrun et al., 2010; Daschmann et al., 2011; Tze et al., 2012, 2016; Chan et al., 2018; Haager et al., 2018; Camacho-Morles et al., 2021; Mata et al., 2021). For example, CB is also negatively correlated with intrinsic goal orientation, task value, learning self-efficacy, intrinsic motivation, degree of effort, learning engagement, use of fine processing strategies, self-regulated learning, and academic performance (Frenzel et al., 2007; Goetz et al., 2007; Tze et al., 2016), and positively correlated with negative emotions and behavioral problems (Pekrun et al., 2010, 2014, 2017). Additionally, CB is suggested to be a major antecedent of later learning weariness and dropping out of school (Sulea et al., 2015). Overall, CB is an important topic worthy of attention from researchers and educators. Additionally, in research on CB, boredom proneness can be considered as a control variable to exclude the individual difference on personality (Wang et al., 2020).

### Effects of Teacher Enthusiasm on CB

Teacher enthusiasm refers to the teachers’ liking for the subject/teaching and the enthusiasm shown in the teaching process; teacher enthusiasm perceived by students is the degree of such positive emotions and display perceived by individual students at the social cognitive level (Keller et al., 2014, 2016). According to the emotional contagion theory (Hatfield et al., 1994), students who experience higher positive emotions from a teacher in class also experience higher levels of positive emotions in classroom learning. Correspondingly, teacher boredom in class, the opposite of teacher enthusiasm, also affects students’ boredom in class (Tam et al., 2020). Kunter et al. (2008) found that teachers who reported higher enthusiasm for mathematics and teaching could provide students with higher levels of cognitive autonomous support, social support, and classroom management. It could be concluded that a high level of enthusiasm for a teacher is associated with some aspects of good teaching quality (Pekrun, 2006; Pekrun et al., 2007, 2010). Additionally, teacher enthusiasm is associated with students’ CB (Cui et al., 2020; Wang et al., 2020). Thus, teachers’ enthusiasm may be an important factor in reducing students’ boredom in class. Based on the previous literature, our first hypothesis is proposed.

Hypothesis 1: Students’ perceived teacher enthusiasm can negatively predict class-related boredom, after controlling for boredom proneness.

### Mediation Effects of Task Value

Students’ PTV may play an intermediary role between their perceived teachers’ enthusiasm and CB. According to the control-value theory, regarding achievement emotion, the external environment may reduce students’ CB through the mediating effect of perceived control and value (Pekrun, 2006; Pekrun et al., 2007). The perception of control and value can be indicated by the students’ perceived task difficulty and task value, respectively. Feelings of worthlessness perceived by students in the learning process represent a significant barrier to their learning success and a major source of negative emotions (Pekrun, 2006). Empirical results show that students’ PTV has a strong predictive effect on their boredom (Pekrun et al., 2010). Bieg et al. (2013) also showed that students’ perceived value is significantly negatively correlated with CB.

Teacher enthusiasm may induce students’ PTV. Teacher enthusiasm can also induce students’ learning value through emotional infection and observation of learning, which is an important means of value induction (Pekrun, 2006). Social learning theory (Bandura, 1977; Frenzel et al., 2007) and the trend of social construction (Wild et al., 1992, 1997) also posit that students’ perceived or observed teacher enthusiasm may affect students’ perception and evaluation of the subject and its task value. Empirical evidence exists that students’ PTE is significantly related to their interest in mathematics (Frenzel et al., 2010), which further predicts individual interest in learning and improves their learning value (Keller et al., 2014). As such, PTE may reduce CB through the mediating effect of students’ PTV. In addition to task value, according to the control-value theory and related empirical research, task difficulty may also affect the relationship between teacher enthusiasm and class boredom (Pekrun, 2006; Cui et al., 2020). Considering the research focus of the present study (i.e., to examine the mediating effect of task value), the influence of task difficulty should be accounted for in the examination. Based on the previous literature, our second hypothesis is proposed.

Hypothesis 2: Students’ perceived task value can mediate the effects of perceived teacher enthusiasm on class-related boredom, after controlling for perceived task difficulty.

Notably, the majority of previous studies have been based on cross-sectional data and could not conclude any causality. The present study intended to collect longitudinal data to explore the mediating mechanism of students’ PTE in alleviating their CB. Longitudinal data can provide a cause-and-effect conclusion with definite direction across time points between variables, and the mediating effect analysis in longitudinal data controls the autoregressive effect of variables, which can provide real-world and reliable research results (Maxwell and Cole, 2007). The longitudinal research design also overcomes the methodological defects of the cross-sectional design in the exploration of causal relations between variables and directional verification of complex mediating mechanisms, thereby compensating for the shortcomings of cross-sectional data at a single time point (being easily affected by the test time and the physical and mental states of college students during testing).

## Materials and Methods

### Participants

The present study was approved by the Research Ethics Committee of the School of Psychology, Beijing Normal University. The participants of this study were first-, second-, and third-year undergraduate students from five colleges in Henan and Shanxi Provinces, China. Originally, 1,729 students completed the survey at the first measurement, but a number of them were dropped out in the process. Overall, data of 1,371 students were included in the analysis (1,050 female, with an average age of 20 years, SD = 1.19).

### Measures

#### Class-Related Boredom (Dependent Variable)

The CB scale from the Achievement Emotions Questionnaire was utilized in the present study (Pekrun et al., 2005; Cui et al., 2017a,b, 2020). The tool contains 11 items, such as “I get bored” and “I find this class fairly dull.” We asked the students to evaluate their experience in the current classroom using a five-point Likert scale ranging from 1 = strongly disagree to 5 = strongly agree. A higher aggregated score indicated higher levels of CB. In the present study, the Cronbach’s alpha was 0.93.

#### Perceived Teacher Enthusiasm (Independent Variable)

We used a three-item instrument to measure PTE, which was used in the study by Keller et al. (2014). These items are the following: “Our teacher in this class teaches with enthusiasm,” “Our teacher in this subject enjoys teaching compared with other courses,” and “Our teacher in this class tries to inspire students about the subject.” The items required the students to evaluate their classroom experience during the day of the survey on a five-point Likert scale, ranging from 1 = strongly disagree to 5 = strongly agree. A higher aggregate score indicates a higher level of PTE. The Cronbach’s alpha for this instrument was 0.90.

#### Perceived Task Value (Mediating Variable)

We assessed students’ PTV using two items: “What I learned in today’s class was useful” and “Compared with what I studied in other courses, what I studied in today’s class was useful” (Tanaka and Murayama, 2014; Cui et al., 2017b). Responses were indicated on a five-point Likert scale ranging from 1 = not at all true of me to 5 = very true of me. A higher aggregate score indicates a higher level of PTV. In the present study, the Cronbach’s alpha was 0.81.

#### Boredom Proneness (Control Variable)

We adapted the 12-item Boredom Proneness Scale-Short Form to measure boredom proneness (Vodanovich et al., 2005). Consistent with previous research (Cui et al., 2017a,b, 2020), we deleted two items to adapt the scale to Chinese culture (i.e., “I find it easy to entertain myself” and “It seems that the same old things are on television or the movies all the time; it’s getting old”). Ten items were maintained, such as “Many things I have to do are repetitive and monotonous.” Responses were indicated on a seven-point Likert scale ranging from 1 = strongly disagree to 7 = strongly agree, which is consistent with the original scale. A higher aggregate score indicates a higher level of boredom proneness. The Cronbach’s alpha was 0.69 for this measure in the present study.

#### Perceived Task Difficulty (Control Variable)

Consistent with previous research, two items were used to assess students’ perceived task difficulty: “Today’s class was hard for me” and “Compared with other courses, today’s class was hard for me” (Tanaka and Murayama, 2014). The participants responded on a five-point Likert scale ranging from 1 = not at all true of me to 5 = very true of me. A higher aggregate score indicates a higher level of perceived task difficulty. In the present study, the Cronbach’s alpha was 0.82.

All the above measure instruments were originally developed in English and translated into Chinese in the present study. The translated version of the scales/items have been used and tested in previous studies (e.g., Cui et al., 2017a; Wang et al., 2020).

### Procedure

The researchers went to the classrooms of various disciplines in these colleges four times, that is, at the second, sixth, tenth, and fourteenth week in a semester (referred to as T1, T2, T3, and T4) to hand out the questionnaires. Informed consent was obtained from all the participants at T1. In each measurement at T1–T4, participants completed the questionnaires approximately 10 min before the end of the class. Overall, 1,371 students completed all the measurements. The missing rate and analysis at T2-T4 was detailed in Section 4.1.

### Data Analysis

We examined the descriptive statistics (mean and SD) and intercorrelations of the variables using PASW statistics for Windows (Version 18, IBM Corp., Armonk, NY, United States). Subsequently, we tested the longitudinal mediation effects of PTE on CB through PTV using a one-way lag model on Mplus 7.0. In the test, perceived task difficulty and boredom proneness were controlled for.

## Results

### Dropout Participants

For the participants, the dropout rate was 7.5% at T2 (*n* = 130/1,729), 8.7% at T3 (*n* = 140/1,599), and 6.0% at T4 (*n* = 88/1,459). The dropout and remaining participants were not significantly different in gender or age. To further test their potential differences on the study variables, independent samples *t*-tests were used to compare the scores of boredom proneness, perceived task difficulty, PTE, CB, and task value in the first round of survey (T1). As shown in Table 1, compared with the participants who completed the whole study, the dropout participants scored significantly higher for the CB and lower for the PTV at the beginning of the study. This result indicated that the dropout of these participants were not completed random. Considering the low rate of dropout participants and the bias to impute the missing data in the longitudinal analysis, only those who completed the whole study were included in the following data analysis.

**TABLE 1:** Differences between the dropout and full-study participants.

Measurement at T1 (*M* ± *SD*)	Dropout participants (*N* = 358)	Full-study participants (*N* = 1,371)	*t*	*p*
Boredom proneness	3.73 ± 0.79	3.64 ± 0.75	1.870	0.062
Perceived task difficulty	2.78 ± 1.01	2.67 ± 1.06	1.724	0.085
Perceived teacher enthusiasm	3.79 ± 0.95	3.85 ± 1.05	–1.155	0.278
Class-related boredom	2.13 ± 0.71	1.92 ± 0.75	4.829	0.000
Perceived task value	3.35 ± 0.95	3.57 ± 0.97	–3.902	0.000

### Descriptive Statistics

Descriptive statistics (i.e., the mean and standard deviation) of the measured variables at T1, T2, T3, and T4 are presented in Table 2. The statistics indicate an increasing tendency of teacher enthusiasm, CB, and task value perceived by students over time.

**TABLE 2:** Participants’ scores for various variables in the four measurements (*N* = 1,371).

	T1	T2	T3	T4
Perceived teacher enthusiasm (*M* ± SD)	3.85 ± 1.05	3.78 ± 0.97	3.77 ± 0.93	3.70 ± 1.00
Class-related boredom (*M* ± SD)	1.92 ± 0.75	2.03 ± 0.78	2.01 ± 0.80	1.96 ± 0.82
Perceived task value (*M* ± SD)	3.57 ± 0.97	3.61 ± 0.88	3.66 ± 0.85	3.62 ± 0.94

### Correlation Analysis

Table 3 lists the correlation coefficients between the variables at the time points of the four measurements. CB at each time point was significantly correlated with PTE and task value, which provided a basis for further analysis of the causation and mediation effects of these variables.

**TABLE 3:** Correlation between variables across the four time points (T1–T4).

	1	2	3	4	5	6	7	8	9	10	11	12
1. PTE-T1	1											
2. PTE-T2	0.23**	1										
3. PTE-T3	0.19**	0.26**	1									
4. PTE-T4	0.11**	0.24**	0.25**	1								
5. CB-T1	−0.18**	−0.15**	−0.20**	−0.10**	1							
6. CB-T2	−0.12**	−0.24**	−0.21**	−0.13**	0.43**	1						
7. CB-T3	−0.09**	−0.20**	−0.30**	−0.16**	0.29**	0.36**	1					
8. CB-T4	–0.05	−0.14**	−0.20**	−0.27**	0.28**	0.31**	0.42**	1				
9. PTV-T1	0.45**	0.15**	0.15**	0.07*	−0.28**	−0.20**	−0.18**	−0.15**	1			
10. PTV-T2	0.15**	0.53**	0.18**	0.21**	−0.19**	−0.29**	−0.20**	−0.19**	0.20**	1		
11. PTV-T3	0.15**	0.19**	0.52**	0.22**	−0.20**	−0.23**	−0.35**	−0.23**	0.23**	0.29**	1	
12. PTV-T4	0.07**	0.16**	0.16**	0.60**	−0.10**	−0.12**	−0.15**	−0.25**	0.08**	0.27**	0.25**	1

*PTE, perceived teacher enthusiasm; CB, class-related boredom; PTV, perceived task value.*

*p < 0.05; **p < 0.01.

### Longitudinal Predictive Effect of PTE on CB

**FIGURE 1:**
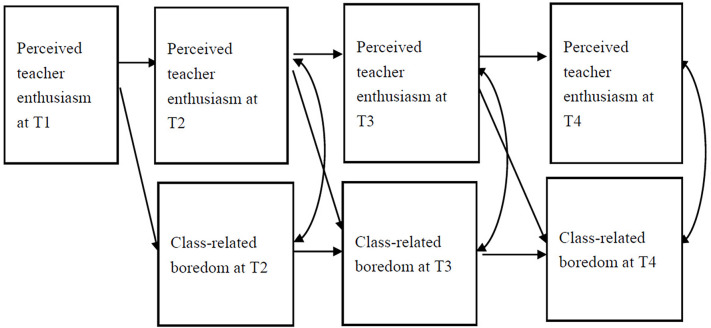
Model diagram of the effect of teacher enthusiasm perceived by students on class-related boredom. To render the model clearly, we do not represent the control variables in the model diagram, but these paths are estimated in the model estimation. The same applies to all other figures.

A one-way lag model was utilized to explore the predictive effect of students’ PTE on their CB. First, we hypothesized that students’ PTE would have a significant predictive effect on CB across time points (see Figure 1). Second, the influence of statistical variables on CB was controlled for by statistical methods (the control variables are not shown in the figure, but the pathways were estimated). This design was expected to exclude the possible effects of a third variable.

**FIGURE 2:**
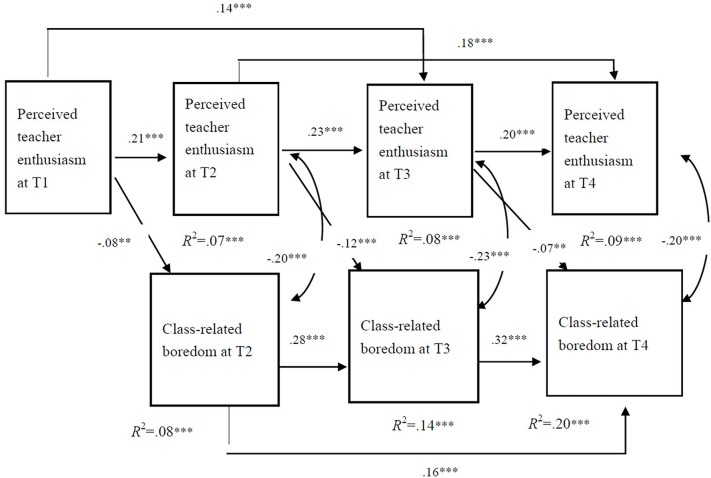
Longitudinal prediction effect of teacher enthusiasm perceived by students on classroom boredom. *p < 0.05; **p < 0.01; ***p < 0.001. The same applies to all other figures.

As shown in Figure 2, in the final model, PTE at T1 significantly predicted the PTE at T2 and T3. PTE-T2 significantly predicted the PTE-T3 and PTE-T4 levels. After controlling for the influence of boredom proneness and perceived task difficulty, we found that students’ PTE at a measuring time point significantly predicted PTE at two time points (except for the predictive effect of T3 on T4), whereas CB at a previous measuring time point significantly predicted CB at two subsequent time points. For the prediction of students’ PTE with respect to their CB across time points, PTE-T1 significantly predicted CB at T2 (Beta = −0.08, *p* < 0.01), PTE-T2 significantly predicted CB-T3 (Beta = −0.12, *p* < 0.001), and PTE-T3 significantly predicted CB-T4 (Beta = −0.07, *p* < 0.01). The fitting indices of the model were as follows: CFI = 0.967, TLI = 0.923, RMSEA (90% CI) = 0.046 [0.033, 0.059], and SRMR = 0.033. The fitting index of this model was acceptable. The model’s path analysis results showed that students’ PTE predicted their experience of CB across time points.

### Mediating Effect Test

**FIGURE 3:**
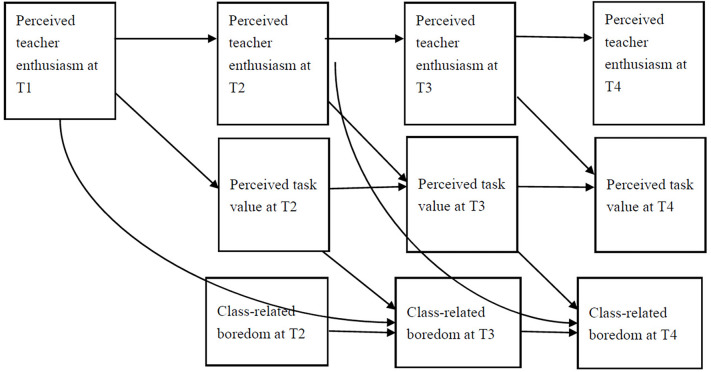
Model diagram of the effect of perceived teacher enthusiasm (PTE) on classroom boredom through the mediation of task value.

Based on the above statistical analysis, we again used a one-way lag model to explore the mediating effect of students’ PTE on their CB through PTV. First, we hypothesized that students’ PTV would have a significant predictive effect across time points (see Figure 3). Second, the influence of statistical variables on CB was controlled by statistical methods (the control variables are not shown in the figure, but these paths were estimated). This design was expected to exclude the possible effects of a third variable.

**FIGURE 4:**
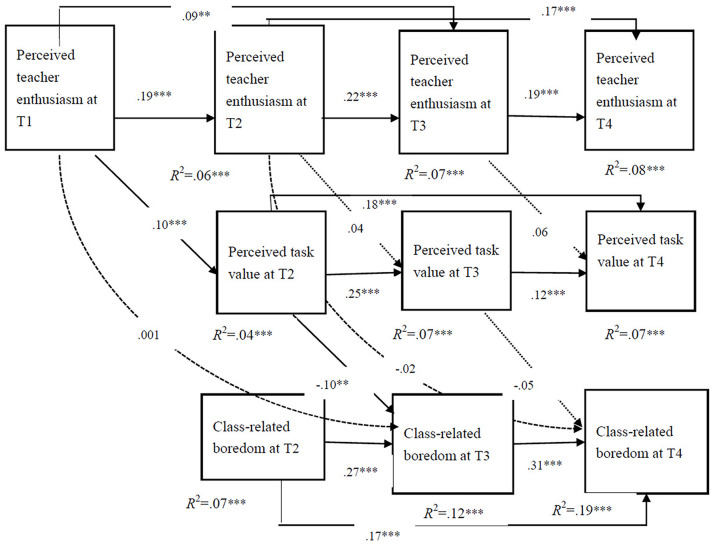
Longitudinal predictive effect of PTE on classroom boredom: perceived task value as intermediary. The solid line represents statistical significance (*p* < 0.05) and the dashed line represents statistical significance (*p* > 0.05).

In the final model (see Figure 4), the model was adjusted according to the fit indices of the data and the hypothesis model. Figure 4 depicts the prediction model of teacher enthusiasm perceived by how students affect experienced classroom boredom through the mediation of PTV and its standardization coefficient.

The change in the model diagram shown in Figure 3 is as follows: PTE-T1 could significantly predict PTE-T2 and PTE-T3, PTE-T2 could significantly predict PTE-T3 and PTE-T4, PTV at T2 could significantly predict PTV-T3 and PTV-T4, and CB-T2 could significantly predict CB-T3 and CB-T4.

The mediating model and its standardized path coefficient of teacher enthusiasm and classroom boredom perceived by students as task values are shown in Figure 4. Individual boredom tendency and task difficulty are important factors that influence students’ CB. To reduce research errors, we controlled for the above two variables (not shown in the model diagram). After controlling for the influence of boredom tendency and task difficulty, we generated the following fitting index of the mediation model: CFI = 0.964, TLI = 0.925, RMSEA (90% CI) = 0.051 [0.043, 0.060], SRMR = 0.050. The model fit was deemed acceptable.

As shown in Figure 4, the prediction of CB-T3 by PTE-T1 was through the complete and significant mediating effect of PTV-T2 (*p* < 0.05). The mediating effect of PTE-T2 on CB-T4 through PTV-T3 was not significant (*p* > 0.05). Based on the significance of the path coefficient in Figure 4, we tested the paths of possible indirect effects and analyzed the indirect effects using a bootstrapping test with deviation correction.

Table 4 presents the results of the bootstrapping tests. The indirect effect of PTE-T1 through PTV-T2 on CB-T3 was significant (−0.010, *p* < 0.05, 95% CI: [−0.016, −0.003]). The indirect effects of PTE-T1 through PTV-T2 and CB-T3 on CB-T4 were significant (−0.003, *p* < 0.05, 95% CI: [−0.005, −0.001]). The total effects of PTE-T1 on CB-T4 were significant (−0.005, *p* < 0.05, 95% CI: [−0.008, −0.002]).

**TABLE 4:** Indirect effects and 95% confidence intervals of the mediating model.

Influence path	Effect size	95% CI
PTE-T1 → PTV-T2 → CB-T3	0.10 × (−0.10) = (−0.010) *	[−0.016, −0.003]
PTE-T1 → PTV-T2 → CB-T3→ CB-T4	0.10 × (−0.10) × 0.31 = (−0.003) *	[−0.005, −0.001]
PTE-T1 → CB-T4 (Total)	−0.005*	[−0.008, −0.002]

## Discussion

Based on the control-value theory (Pekrun, 2006; Pekrun et al., 2007), we used longitudinal data to explore the predictive effect of teachers’ enthusiasm on college students’ CB and the mediating effect of students’ PTV. The results shed light on the relationship between teachers’ enthusiasm and students’ CB, as well as on the role of teachers’ enthusiasm and the appropriate means to reduce students’ CB. The two hypotheses have been supported.

### Students’ PTE Predicts Their CB

In this study, college students from five universities were measured four times during a semester. The following results supported our first hypothesis: PTE-T1 significantly predicted CB-T2, PTE-T2 significantly predicted CB-T3, and PTE-T3 significantly predicted CB-T4. The students’ PTE in the previous month significantly negatively predicted their CB in the following month; that is, college students’ PTE played a role in reducing their CB, which confirmed the research hypothesis that college students’ PTE could reduce their CB across time points.

After controlling for the effects of auto regression and other factors on college students’ CB, we found that college students’ PTE in the preceding month could predict their CB significantly and negatively in the succeeding month, confirming that college students’ PTE played an important role in reducing their CB. Our results are consistent with those of previous studies (Goetz et al., 2006; Keller et al., 2014; Kim and Schallert, 2014). Goetz et al. (2006) considered PTE as an important teaching variable in Latin class in order to investigate the relationship between it and students’ classroom emotion; their results showed a high correlation between PTE and students’ boredom.

### Mediating Role of PTV

The results show that college students’ PTV played a significant longitudinal mediating role between their PTE and CB, supporting the second hypothesis. The participants who perceived higher teacher enthusiasm in the first month also perceived higher task value in the second month and experienced lower CB in the third month. Thus, college students’ PTE may play a role in improving students’ PTV and reducing their CB. The participants’ PTE in the second month could not predict their CB in the fourth month through the mediating effect of the task value perceived in the third month. Students’ PTE at the beginning of the semester (the first month) had a more significant longitudinal predictive effect on their PTV and CB, compared with the middle and end of the semester. One possible reason is that with the approaching final examination, college students perceived a high level of task value and a lower level of CB in the fourth month (see Figure 4 and Table 2).

The longitudinal mediating effect of students’ PTV between their PTE and CB revealed in this study can be explained by previous theories and empirical studies. Pekrun (2006) stated that value induction can propel students’ perceived value to a higher level, and that promoting teachers’ enthusiasm is an important way to induce value. Pekrun (2006) further found that the most important reason for students’ CB is the perception that their learning tasks are worthless. Therefore, teacher enthusiasm can play the role of value induction to enhance the value of tasks and reduce students’ CB. Similar studies have found a significant predictive role of perceived value on boredom (Ekatushabe et al., 2021; Forsblom et al., 2021).

### Implications

Using longitudinal data at four time points, this study confirmed that teacher enthusiasm can help reduce students’ CB through the mediation of students’ PTV. The present results showed that college students perceived more task value from lessons with high teacher enthusiasm, and ultimately experienced reduced CB. Researchers and primary educators should recognize that teachers’ enthusiasm plays an important role in reducing students’ CB. In terms of practical implications, our study identified a new path for reducing college students’ CB. Regardless of course variation and quality of teaching, as long as teachers have high enthusiasm for teaching, as well as for the subjects and students, which can be perceived by students, then CB can be reduced because the students can perceive more task value in the class. The path of increasing the value of tasks through teachers’ enthusiasm, thereby reducing students’ CB, is simple, efficient, and has multiple effects. Education researchers and practitioners need to pay attention to promoting teachers’ enthusiasm for the subjects and teaching.

### Limitations and Future Research

The present study had some limitations. Firstly, although we adopted a longitudinal study design to explore the causal relations among the variables, such causal relations could not be deduced in a strict sense (i.e., no mediation between T2 enthusiasm and T4 boredom). In our longitudinal design, the measurement was conducted four times within a semester. At T4, the occasion was very close to the end of the semester and students were in a state quite different from that in the normal time. College students had pressure to pass their final examinations and their academic emotion might on a special level. In the future, the time points of measurements should be carefully selected and experimental studies, intervention studies, and other paradigms could be used to further test the results of our study.

Secondly, the non-random dropout participants might lead to bias and we should take caution to generalize the results. Specifically, our survey was taken place 10 min before the end of the students’ learning of a class, and hence students would miss the survey if they did not attend the class. In other words, students who had a high rate of absenteeism in a class, who usually experienced much boredom and little value in class, tend to drop out from the present study. Therefore, the findings of the present study might not be able to generalize to these students.

Thirdly, the participants were from various disciplines in five colleges, but we did not measured information of their classes and analyzed data of different classes/colleges together. Theoretically, it will be ideal to construct a multilevel model when the differences among different classes are significant. However, previous research based on cross-sectional designs had supported the universal mediating effects of task value between teacher enthusiasm and CB cross different universities and classes (e.g., Wang et al., 2020). This means that the analysis on the whole sample would rarely bias the results, whereas future research can improve sampling strategies and utilize multilevel models in analysis.

Finally, according to the control-value theory, in addition to PTE, task value and task difficulty, teaching quality, students’ achievement goals, and academic performance may play a role in reducing students’ CB (Pekrun, 2006; Pekrun et al., 2007). These additional variables were not included in our study and should be considered in future studies to provide a clearer picture of the effects of teachers’ enthusiasm on student learning. As to task difficulty, considering the research aim, it was controlled for in the present study to ease the data analysis and interpretation. In future research, the effect of task difficulty could be further explored.

## Conclusion

Our study expands upon existing knowledge regarding the longitudinal relationship among teacher enthusiasm, task value, and CB. Our findings are novel and insightful, both theoretically and practically. We not only clarify that college students’ PTE negatively predicted their CB across time points but also provide evidence on the role of students’ PTV as a longitudinal mediator in this relationship. Our results suggest that college students’ PTV mediated the dampening effect of PTE on CB across time points. To this end, the present study offers an important foundation for future research in this area.

## Data Availability Statement

The datasets generated during and/or analyzed during the current study are available from the corresponding author on reasonable request.

## Ethics Statement

The studies involving human participants were reviewed and approved by The Research Ethics Committee of School of Psychology, Beijing Normal University. The Ethics Committee waived the requirement of written informed consent for participation. Students’ return of the completed questionnaire at T1 was accepted as an indication of their consent to participate in the present study.

## Author Contributions

G-yC, CW, and Y-jH contributed to the conception construction, design of the study, analysis, and interpretation of the data. J-yC, CZ, and XZ contributed to the collection of the data. G-yC and Y-jH wrote the original manuscript. CW made critical revisions. All authors approved the final manuscript for publication.

## Conflict of Interest

The authors declare that the research was conducted in the absence of any commercial or financial relationships that could be construed as a potential conflict of interest.

## Publisher’s Note

All claims expressed in this article are solely those of the authors and do not necessarily represent those of their affiliated organizations, or those of the publisher, the editors and the reviewers. Any product that may be evaluated in this article, or claim that may be made by its manufacturer, is not guaranteed or endorsed by the publisher.

## References

[B1] Bandura A. (1977). *Social Learning Theory.* Englewood Cliffs: Prentice Hall.

[B2] Bieg M. Goetz T. Hubbard K. (2013). Can I master it and does it matter? An intraindividual analysis on control-value antecedents of trait and state academic emotions. *Learn. Individ. Differ.* 28 102–108. 10.1016/j.lindif.2013.09.006

[B3] Britton A. Shipley M. J. (2010). Bored to death? *Int. J. Epidemiol.* 39 370–371. 10.1093/ije/dyp404 20361429

[B4] Camacho-Morles J. Slemp G. R. Loderer K. Hou H. Oades L. (2021). Activity Achievement Emotions and Academic Performance: a Meta-analysis. *Educ. Psychol. Rev.* 10.1007/s10648-020-09585-3

[B5] Chan C. S. van Tilburg W. A. P. Igou E. R. Poon C. Y. S. Tam K. Y. Y. Wong V. U. T. (2018). Situational meaninglessness and state boredom: cross-sectional and experience-sampling findings. *Motiv. Emot.* 42 555–565. 10.1007/s11031-018-9693-3

[B6] Cui G. Lan X. Zhang X. Hu Y. Wang C. (2020). The association between teacher enthusiasm and students’ class-related boredom: a multilevel study. *Curr. Psychol.* 11:323. 10.1007/s12144-020-00890-2

[B7] Cui G. Yao M. Zhang X. (2017a). Can nursing students? perceived teacher enthusiasm dampen their class-related boredom during theoretical lessons? A cross-sectional study among Chinese nursing students. *Nurse Educ. Today* 53 29–33. 10.1016/j.nedt.2017.04.003 28426998

[B8] Cui G. Yao M. Zhang X. (2017b). The dampening effects of perceived teacher enthusiasm on class-related boredom: the mediating role of perceived autonomy support and task value. *Front. Psychol.* 8:400. 10.3389/fpsyg.2017.00400 28367134 PMC5356221

[B9] Daschmann E. C. Goetz T. Stupnisky R. H. (2011). Testing the predictors of boredom at school. Development and validation of the Precursors to Boredom Scales. *Br. J. Educ. Psychol.* 81 421–440. 10.1348/000709910X526038 21770913

[B10] Ekatushabe M. Kwarikunda D. Muwonge C. M. Ssenyonga J. Schiefele U. (2021). Relations between perceived teacher’s autonomy support, cognitive appraisals and boredom in physics learning among lower secondary school students. *Int. J. STEM Educ.* 8:8. 10.1186/s40594-021-00272-5

[B11] Forsblom L. Peixoto F. Mata L. (2021). Perceived classroom support: longitudinal effects on students’ achievement emotions. *Learn. Individ. Differ.* 85:101959. 10.1016/j.lindif.2020.101959

[B12] Frenzel A. C. Goetz T. Pekrun R. Watt H. M. G. (2010). Development of Mathematics Interest in Adolescence: influences of Gender, Family, and School Context. *J. Res. Adolesc.* 20 507–537. 10.1111/j.1532-7795.2010.00645.x

[B13] Frenzel A. C. Pekrun R. Goetz T. (2007). Perceived learning environment and students’ emotional experiences: a multilevel analysis of mathematics classrooms. *Learn Instr.* 17 478–493. 10.1016/j.learninstruc.2007.09.001

[B14] Goetz T. Frenzel A. C. Pekrun R. Hall N. C. Lüdtke O. (2007). Between- and within-domain relations of students’ academic emotions. *J. Educ. Psychol.* 99 715–733. 10.1037/0022-0663.99.4.715

[B15] Goetz T. Pekrun R. Hall N. Haag L. (2006). Academic emotions from a social-cognitive perspective: antecedents and domain specificity of students’ affect in the context of Latin instruction. *Br. J. Educ. Psychol*. 76 289–308. 10.1348/000709905X42860 16719965

[B16] Haager J. S. Kuhbandner C. Pekrun R. (2018). To Be Bored or Not To Be Bored—How Task-Related Boredom Influences Creative Performance. *J. Creat. Behav.* 52 297–304. 10.1002/jocb.154

[B17] Harris M. B. (2000). Correlates and characteristics of boredom proneness and boredom. *J. Appl. Soc. Psychol.* 30 576–598. 10.1111/j.1559-1816.2000.tb02497.x

[B18] Hatfield E. Cacioppo J. T. Rapson R. L. (1994). *Emotional Contagion.* New York: Cambridge University Press.

[B19] Keller M. M. Goetz T. Becker E. S. Morger V. Hensley L. (2014). Feeling and showing: a new conceptualization of dispositional teacher enthusiasm and its relation to students’ interest. *Learn Instr.* 33 29–38. 10.1016/j.learninstruc.2014.03.001

[B20] Keller M. M. Hoy A. W. Goetz T. Frenzel A. C. (2016). Teacher enthusiasm: reviewing and redefining a complex construct. *Educ. Psychol. Rev.* 28 743–769. 10.1007/s10648-015-9354-y

[B21] Kim T. Schallert D. L. (2014). Mediating effects of teacher enthusiasm and peer enthusiasm on students’ interest in the college classroom. *Contemp. Educ. Psychol.* 39 134–144. 10.1016/j.cedpsych.2014.03.002

[B22] Kunter M. Tsai Y.-M. Klusmann U. Brunner M. Krauss S. Baumert J. (2008). Students’ and mathematics teachers’ perceptions of teacher enthusiasm and instruction. *Learn Instr.* 18 468–482. 10.1016/j.learninstruc.2008.06.008

[B23] Maher P. J. Martin D. G. van Tilburg W. A. P. Igou E. R. Moynihan A. B. (2018). Bored like Hell: religiosity reduces boredom and tempers the quest for meaning. *Emotion* 19 255–269. 10.1037/emo0000439 29697990

[B24] Martz M. E. Schulenberg J. E. Patrick M. E. Kloska D. D. (2018). “I Am So Bored!”: prevalence Rates and Sociodemographic and Contextual Correlates of High Boredom Among American Adolescents. *Youth Soc.* 50 688–710. 10.1177/0044118X15626624

[B25] Mata L. Monteiro V. Peixoto F. Santos N. N. Sanches C. Gomes M. (2021). *Emotional Profiles Regarding Maths Among Primary School Children – A two-year Longitudinal Study.* Germany: Springer. 10.1007/s10212-020-00527-9

[B26] Maxwell S. E. Cole D. A. (2007). Bias in cross-sectional analyses of longitudinal mediation. *Psychol. Methods* 12 23–44. 10.1037/1082-989x.12.1.23 17402810

[B27] Pekrun R. (2006). The control-value theory of achievement emotions: assumptions, corollaries, and implications for educational research and practice. *Educ. Psychol. Rev.* 18 315–341. 10.1007/s10648-006-9029-9

[B28] Pekrun R. Frenzel A. Goetz T. Perry R. P. (2007). “The control-value theory of achievement emotions: an integrative approach to emotions in education” in *Emotion in Education.* eds Schutz P. A. Pekrun R. (San Diego: Academic Press). 13–36.

[B29] Pekrun R. Goetz T. Perry R. (2005). *Achievement Emotions Questionnaire (AEQ). User’s Manual.* Munich: University of Munich.

[B30] Pekrun R. Goetz T. Daniels L. M. Stupnisky R. H. Perry R. P. (2010). Boredom in achievement settings: control-value antecedents and performance outcomes of a neglected emotion. *J. Educ. Psychol.* 102 531–549. 10.1037/a0019243

[B31] Pekrun R. Hall N. C. Goetz T. Perry R. P. (2014). Boredom and academic achievement: testing a model of reciprocal causation. *J. Educ. Psychol.* 106 696–710. 10.1037/a0036006

[B32] Pekrun R. Lichtenfeld S. Marsh H. W. Murayama K. Goetz T. (2017). Achievement Emotions and Academic Performance: longitudinal Models of Reciprocal Effects. *Child Dev.* 88 1–18. 10.1111/cdev.12704 28176309

[B33] Schwartze M. M. Frenzel A. C. Goetz T. Marx A. K. G. Reck C. Pekrun R. (2020). Excessive boredom among adolescents: a comparison between low and high achievers. *PLos One* 15:e0241671. 10.1371/journal.pone.0241671 33152022 PMC7644046

[B34] Sharp J. G. Hemmings B. Kay R. Sharp J. C. (2019). Academic boredom and the perceived course experiences of final year Education Studies students at university. *J. Furth. High. Educ.* 43 601–627. 10.1080/0309877X.2017.1386287

[B35] Sulea C. van Beek I. Sarbescu P. Virga D. Schaufeli W. B. (2015). Engagement, boredom, and burnout among students: basic need satisfaction matters more than personality traits. *Learn. Individ. Differ.* 42 132–138.

[B36] Tam K. Y. Y. Poon C. Y. S. Hui V. K. Y. Wong C. Y. F. Kwong V. W. Y. Yuen G. W. C. (2020). Boredom begets boredom: an experience sampling study on the impact of teacher boredom on student boredom and motivation. *Br. J. Educ. Psychol.* 90 124–137. 10.1111/bjep.12309 31342514

[B37] Tam K. Y. Y. van Tilburg W. A. P. Chan C. (2021). What is boredom proneness? A comparison of three characterizations. *J. Pers.* 10.1111/jopy.12618 33484603

[B38] Tanaka A. Murayama K. (2014). Within-person analyses of situational interest and boredom: interactions between task-specific perceptions and achievement goals. *J. Educ. Psychol.* 106 1122–1134. 10.1037/a0036659

[B39] Tze V. M. C. Daniels L. M. Klassen R. M. (2016). Evaluating the relationship between boredom and academic outcomes: a meta-analysis. *Educ. Psychol. Rev.* 28 119–144. 10.1007/s10648-015-9301-y

[B40] Tze V. M. C. Klassen R. M. Daniels L. M. Li J. C.-H. Zhang X. (2012). A cross-cultural validation of the learning-related boredom scale (LRBS) With Canadian and Chinese college students. *J. Psychoeduc. Assess.* 31 29–40.

[B41] Vodanovich S. J. Wallace J. C. Kass S. J. (2005). A confirmatory approach to the factor structure of the boredom proneness scale: evidence for a two-factor short form. *J. Pers. Assess.* 85 295–303. 10.1207/s15327752jpa8503_05 16318568

[B42] Wang C. Hu Y. Zhang X. Wang J. Cui G. Cui G. (2020). Stability of the mitigating effect of students’ perceived teacher enthusiasm on class-related boredom: moderating role of boredom proneness and perceived task difficulty. *Int. J. Environ. Res. Public Health* 17:2645. 10.3390/ijerph17082645 32290592 PMC7216052

[B43] Wild T. C. Enzle M. E. Hawkins W. L. (1992). Effects of perceived extrinsic versus intrinsic teacher motivation on student reactions to skill acquisition. *Pers. Soc. Psychol. Bull.* 18 245–251. 10.1177/0146167292182017

[B44] Wild T. C. Enzle M. E. Nix G. Deci E. L. (1997). Perceiving others as intrinsically or extrinsically motivated: effects on expectancy formation and task engagement. *Pers. Soc. Psychol. Bull.* 23 837–848. 10.1177/014616729723800

